# Zika Virus: A Neurotropic Warrior against High-Grade Gliomas—Unveiling Its Potential for Oncolytic Virotherapy

**DOI:** 10.3390/v16040561

**Published:** 2024-04-03

**Authors:** María-Angélica Calderón-Peláez, Silvia Juliana Maradei Anaya, Ingrid Juliana Bedoya-Rodríguez, Karol Gabriela González-Ipuz, Daniela Vera-Palacios, Isabella Victoria Buitrago, Jaime E. Castellanos, Myriam L. Velandia-Romero

**Affiliations:** 1 Virology Group, Vice-Chancellor of Research, Universidad El Bosque, Bogotá 110121, Colombia; mcalderon@unbosque.edu.co (M.-A.C.-P.); smaradei@unbosque.edu.co (S.J.M.A.); castellanosjaime@unbosque.edu.co (J.E.C.); 2 Semillero ViroLogic 2020–2022, Virology Group, Vice-Chancellor of Research, Universidad El Bosque, Bogotá 110121, Colombia

**Keywords:** glioblastoma multiforme, neurotropism, oncolytic virus, ZIKV

## Abstract

Gliomas account for approximately 75–80% of all malignant primary tumors in the central nervous system (CNS), with glioblastoma multiforme (GBM) considered the deadliest. Despite aggressive treatment involving a combination of chemotherapy, radiotherapy, and surgical intervention, patients with GBM have limited survival rates of 2 to 5 years, accompanied by a significant decline in their quality of life. In recent years, novel management strategies have emerged, such as immunotherapy, which includes the development of vaccines or T cells with chimeric antigen receptors, and oncolytic virotherapy (OVT), wherein wild type (WT) or genetically modified viruses are utilized to selectively lyse tumor cells. In vitro and in vivo studies have shown that the Zika virus (ZIKV) can infect glioma cells and induce a robust oncolytic activity. Consequently, interest in exploring this virus as a potential oncolytic virus (OV) for high-grade gliomas has surged. Given that ZIKV actively circulates in Colombia, evaluating its neurotropic and oncolytic capabilities holds considerable national and international importance, as it may emerge as an alternative for treating highly complex gliomas. Therefore, this literature review outlines the generalities of GBM, the factors determining ZIKV’s specific tropism for nervous tissue, and its oncolytic capacity. Additionally, we briefly present the progress in preclinical studies supporting the use of ZIKV as an OVT for gliomas.

## 1. Introduction

Zika virus (ZIKV) is a flavivirus with a single-stranded positive-sense RNA genome [1] that is primarily transmitted through the bite of female mosquitoes of the Aedes genus (aegypti, albopictus). However, it can also be transmitted through parenteral routes (from mother to child), sexual contact, or blood transfusion [2,3]. Zika fever is asymptomatic in 80% of cases or may present with self-limiting and nonspecific symptoms (similar to those caused by other flaviviruses such as dengue virus). Despite ZIKV’s tropism for nervous tissue cells, it has been shown that this virus can also infect other cell types, including keratinocytes, dermal fibroblasts, and dendritic cells [4]. Importantly, during the ZIKV outbreak in the Americas between 2015–2016, it was reported that ZIKV has the capability to pass through highly selective cellular barriers, such as the placental barrier and the blood–brain barrier (BBB), infecting neurons, astrocytes, and both differentiated and immature microglial cells, among others [5]. This neurotropism explains the damage induced by the virus in the nervous tissue, such as the presentation of microcephaly in individuals infected during their fetal development [6] or Guillain–Barré Syndrome in adults [7]. Microcephaly, among other brain damage in fetuses, is partly due to the lytic effect of the virus on the nervous tissue cells, including glial stem cells, suggesting that ZIKV is a lytic virus that could be capable of inducing the lysis of complex brain tumors deriving from undifferentiated and anaplastic glial cells, such as glioblastoma multiforme (GBM) [8,9].

Tumors of the nervous tissue, known as gliomas, originate from glial cells (oligodendrocytes, astrocytes, and microglia) or their precursors. They are among the most complex cancers, not only due to their poor prognosis but also because of the direct repercussions on the quality of life and cognitive function of patients [10,11]. The World Health Organization (WHO) classifies gliomas into four histological grades (I–IV) based on increasing levels of undifferentiation, anaplasia, and aggressiveness. Grade III tumors (anaplastic variants of astrocytoma, oligodendroglioma, and oligoastrocytoma) and Grade IV tumors, referred to as “high-grade” or “malignant”, are characterized by a rapid evolution of the disease and a fatal outcome [10,11,12]. Within Grade IV tumors, GBM represents between 12% and 15% of intracranial neoplasms, being the most aggressive and frequent in the central nervous system (CNS). Following the diagnosis, some patients have a median survival rate of 14 to 15 months [10], of which only 33% survive, and about 5% of these survive for an additional five years after tumor resection [13].

**Figure 1:**
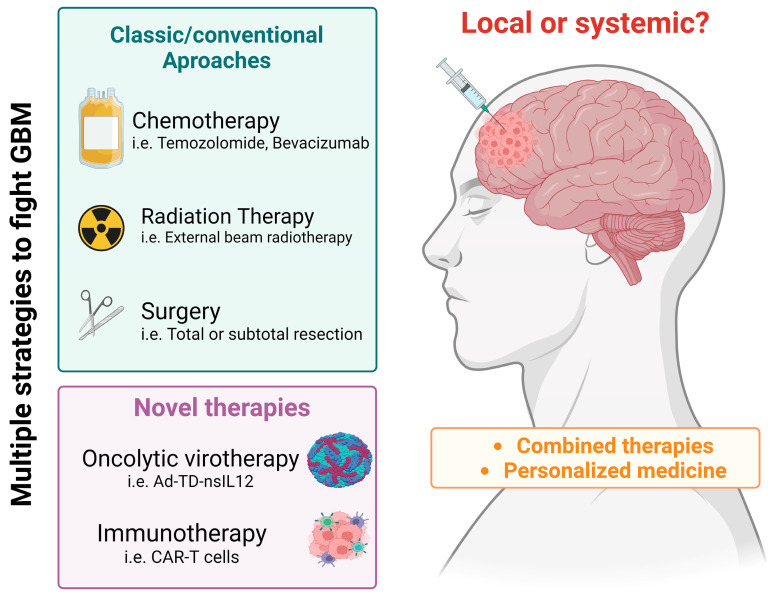
Scheme of the treatment strategies for GBM. Image created using Biorender.com (last accessed on 29 January 2024).

Currently, the first line of treatment for GBM involves surgical resection of the largest possible tumor area, combined with radiotherapy and/or chemotherapy [11]. The effectiveness of these treatments is, however, very limited, leading to the study and proposition of better-targeted therapies designed to enhance the pro-apoptotic effects on tumor cells; molecular biology and immunology have played a significant role in the development of such therapies, becoming a hope in increasing the survival rates of patients [14] (Figure 1). This has led to the discovery and application of lytic-capable viruses as destructive agents of tumor cells, known as oncolytic viruses or “OVs” [15]. Within this select group are some adenoviruses (AdVs), the Herpes Simplex virus (HSV), and some rotaviruses (RVs) [16], several of which have already been approved as therapeutic candidates for use in specific tumors, or which are still undergoing clinical trials [17]. Interestingly, some researchers have discussed ZIKV as a potential OV for controlling GBM due to its ability to infect immature or undifferentiated neuro-glial cells, which are typical of these tumors [18].

## 2. Biology of GBM

GBMs are diffusely infiltrating tumors characterized by a high degree of undifferentiation, typically originating in the white matter and exhibiting significant heterogeneity in shape and appearance, hence the term “multiforme” [19]. While they commonly manifest in the cortex, GBMs have also been identified in diverse brain regions such as the brainstem, thalamus, cerebellum, and spinal cord [11]. Although metastasis is infrequent, its occurrence may impact various extracranial sites, including bones, lungs, lymph nodes, neck, and liver, with liver metastasis associated with the poorest prognosis and outcome [20]. Importantly, the clinical presentation of GBM varies, being contingent upon the tumor’s location and size. Patients often exhibit symptoms related to the functional impairment of the affected cerebral area and an increase in intracranial pressure. For instance, tumors in the frontal or temporal lobe or in the corpus callosum can induce subtle symptoms like cognitive dysfunction, mood disorders, fatigue, and mild memory disorders [10,19]. While GBM can develop at any age, their highest incidence is observed between 50 and 60 years. Notably, GBM ranks as the third leading cause of death in individuals aged 15 to 34 years [10,20].

Histologically, GBM exhibits a diverse cellular composition characterized by cells displaying high morphological variability. The presence of pleomorphic and multinucleated cells with marked mitotic activity, extensive angiogenesis, and endothelial hyperplasia are distinctive features. In addition, histological sections commonly reveal intravascular microthrombi and extensive ischemic or palisade-like necrosis, confirming the diagnosis [21,22]. Definitive diagnosis hinges on the histopathological examination of the excised tumor, emphasizing the identification of infiltration and positivity for glial fibrillary acidic protein (GFAP), coupled with significant pleomorphism, rapid mitotic activity, microvascular proliferation, and necrosis [21].

Presently, two morphologically identical subtypes of GBMs are recognized, referred to as GBM-I and -II [23]. GBM-I is distinguished as a primary or de novo glioblastoma, more prevalent in older adults (>60 years), while GBM-II develops from a low-grade astrocytoma undergoing anaplastic transformation, primarily affecting young individuals in the frontal lobe, and constituting less than 10% of these tumors. Significantly, both entities differ, evolving through distinct molecular pathways [23]. For instance, GBM-I exhibits overexpression of the epidermal growth factor receptor (EGFR) and mutations in the phosphatase and tensin homolog (PTEN), alongside loss of chromosome 10q. Conversely, GBM-II is characterized by mutations in isocitrate dehydrogenase 1 (IDH1), p53, loss of chromosome 19q, and the absence of regulation of the antiapoptotic protein Bcl-xL. These variances impact the response of GBM to treatment [23,24,25]. GBM-I responds to therapies targeting EGFR, such as Temozolomide (TMZ), and monoclonal antibodies like bevacizumab [25], while GBM-II is susceptible to chemoradiotherapy and Bcl-xL inhibitors [24].

The genesis of GBM involves molecular alterations in tumor suppressor genes (TSGs), oncogenes, and DNA-repairing genes [10]. The most common alterations are associated with the P53 signaling pathways, the tyrosine kinase receptor/Ras/phosphoinositide 3-kinase pathway, and the retinoblastoma pathway [23]. These alterations result in uncontrolled cell proliferation and increased survival, facilitated by the ability of these cells to escape the G2/M cell-cycle checkpoint blockade, thereby avoiding apoptosis [10].

As previously mentioned, the primary treatment for GBM involves the surgical resection of the largest possible tumor area, combined with radiotherapy and/or chemotherapy [11]. However, these treatments pose therapeutic challenges: they lack specificity in distinguishing between cancerous and normal cells, and the therapy’s effectiveness is closely linked to the patient’s age, radiation dose, and tumor volume [10]. Moreover, over time, nearly all tumors recur, becoming more aggressive and less responsive to treatment. Recurrences often occur in new brain areas, making a second surgical resection challenging or impossible (only 20–30% of recurrent GBM (GBR) are operable).

Most recurrences are local, with about two-thirds of tumors re-emerging within 2 cm of the initial margin. They involve larger areas of necrotic tissue with fewer tumor cells compared with their primary counterparts. However, a third of GBRs appear far from the initial tumor area; in a different lobe, in the contralateral hemisphere, or even in the infratentorial region. Currently, there is no standard treatment for GBR, and patients succumb to the malignant disease 12 to 15 months after the initial diagnosis [26].

Therefore, the exploration of alternative therapies has become not only necessary but urgent, prompting the consideration and development of various drugs and molecules to complement surgical resection. One notable example is the development of therapies targeting tumor angiogenesis, such as Bevacizumab, a targeted therapeutic agent that binds to the vascular endothelial growth factor (VEGF) [19]. Another recent treatment modality is photodynamic therapy, which exposes the tumor to laser light in the range of 400 to 900 nm. This technique is primarily employed to identify residual tumors or surgical resection boundaries [27].

Innovative approaches also include the use of 22-basepair non-coding RNAs (ncRNAs) that regulate gene expression by binding to complementary sequences in messenger RNA (mRNA), thereby silencing protein translation. These ncRNAs are considered epigenetic effectors and hold promise in GBM treatment [28]. Additionally, a recent advancement involves the use of replicative viruses in tumor cells, known as OVs, which induce a proinflammatory response and cause cell lysis [29]. These diverse therapeutic strategies represent promising avenues for improving the treatment landscape of GBM.

## 3. The Oncolytic Viruses

OVs are either attenuated, mutated, or naturally occurring viruses that have been designed to selectively replicate in tumor cells, causing their death while sparing normal cells and preserving the tissue architecture. The concept of using viruses as OVs has deep roots, stemming from evidence of tumor regressions during or after naturally acquired systemic viral infections [29]. Early clinical references to OVs date back to the early 20th century, with case reports noting remission of malignant tumors, particularly leukemias or lymphomas, following viral infections or the application of attenuated viruses as vaccination strategies. This underscored the viruses’ potential to elicit a prolonged antitumor response [30].

A significant milestone occurred in 1998 when a mutant form of HSV-1, named G207, was employed to treat malignant glioma. This marked the inception of utilizing selectively replicating viruses in cancer cells. Subsequently, the search for naturally occurring OVs and those experimentally modified or designed gained momentum [29]. The first OV to demonstrate positive results in phase III clinical trials was a derivative of HSV, known as Talimogene laherparepvec (T-VEC), which gained the United States Food and Drug Administration Agency (FDA) approval in 2015 for treating advanced metastatic melanoma. It was later approved in Europe in 2016 [31]. Several countries, including China and Japan, have also approved OVs for tumor treatments. For instance, the modified AdV (H101) is used to treat head and neck cancer in China (approved in 2005), and the mutated HSV virus (G47) is employed in Japan for GBM treatment, approved in 2009 [29].

OVs can exhibit natural or acquired tropism, selectively infecting tumor cells, with the latter achieved through genetic engineering [32]. A prime example is the Edmonston strain of the measles virus (MV), which needs the expression of CD46 on the surface of malignant cells for virus recognition and entry. Given the overexpression of CD46 in cancer cells, MV exhibits high specificity for infecting these cells [33,34]. OVs possess the natural or induced ability to replicate efficiently within malignant cells, promoting apoptosis and autophagy [32]. This process facilitates the release of new viral particles into the extracellular environment, enabling the infection of neighboring cancer cells. Consequently, this mechanism allows the OV to spread within the tumor mass, reaching a broader area of cancer cells and potentially being delivered to distant metastatic tumor cells [29]. Another example are reoviruses (REOs) which not only demonstrate tropism for a diverse range of tumor cells but also exhibit high replication rates inside them. This capability arises from the virus’s natural tendency to replicate easily in cells with dysregulated growth-factor signaling cascades, characteristic of tumor cells. REOs can directly lyse tumor cells, particularly those with defects in antiviral signal transduction mediated by PKR, an interferon (IFN)-induced kinase crucial in the innate antiviral immune response. Additionally, REOs can induce natural killer (NK) cell-mediated cytotoxicity and T cell-mediated cytotoxicity, amplifying the host’s antitumor response [29,35].

Significantly, the rational selection of a virus for use as an oncolytic agent must consider various factors, including the type and location of the tumor to be treated, among other critical characteristics [36,37]. Several key considerations reviewed elsewhere [36,37,38,39] come into play, such as:

(i) Tumor Tropism: the virus’s ability to selectively target and infect tumor cells.
(ii) Infection of Non-tumoral Cells: whether the virus can infect non-tumoral cells or is specific to cancer cells.
(iii) Encoding Therapeutic Transgenes: the capacity of the virus to carry and express therapeutic transgenes.
(iv) Replication and Viral Progeny Production: the ability of the virus to replicate and generate viral progeny, including viremia in blood and tissues.
(v) Virus Infiltration into Tissues: the extent to which the virus can infiltrate tissues.
(vi) Viral Evasion of the Immune Response: the virus’s ability to evade the host immune response.
(vii) Minimizing Adverse Effects: strategies aimed at minimizing potential adverse effects.
(viii) Virus Pathogenicity and Immunogenicity: the virus’s inherent pathogenicity and its potential to induce an immune response.

Considering these crucial criteria is paramount for ensuring the safe and effective application of OVs in antitumor therapy [36,39]. Table 1 shows some of the studied OVs that are currently part of clinical trials, their characteristics, and the types of tumors to which they have been applied.

## 4. New Therapies for GBM Treatment

GBM is characterized by its cellular and molecular complexities, inherent treatment resistance, and high recurrence rates, which often leads to rapid neurological deterioration and a decline in overall patient health, ultimately culminating in fatality. Recognizing the need for more effective treatments, efforts have been directed toward correlating molecular characteristics and GBM subtypes with the prognosis and treatment response to tailor individual therapeutic approaches [51].

The typification of genetic variants continues to be recommended for case stratification and the establishment of management plans [11]. Notably, the methylation status of the promoter region of the MGMT gene (O-6-Methylguanine-DNA-methyltransferase) stands out as a well-established prognostic factor and a predictor of the response to TMZ treatment, the widely used chemotherapeutic agent for GBM [52,53]. Loss-of-function mutations in IDH1 (cytoplasmic and peroxisomal) and IDH2 (mitochondrial) have been identified in 50 to 80% of low-grade gliomas, and at least 75% of GBM-II cases. These mutations are associated with a more favorable prognosis, increased progression-free survival, and improved response to chemotherapy [54]. Additionally, variations in the copy number or amplification of gene expression involved in cell-cycle regulatory processes have emerged as prognostic factors or predictors of response to specific GBM treatments. Notably, the overexpression and constitutive activation of platelet-derived growth factor (PDGF) and its alpha receptor (PDGFRα) are prevalent in the majority of GBM cases. Research into the therapeutic targeting of signaling pathways related to PDGF and PDGFRα is actively underway [55]. These advancements signify a paradigm shift toward more personalized and targeted therapeutic strategies in the ongoing battle against GBM.

Despite advances in more aggressive and targeted treatments, approximately 70% of GBM patients experience disease progression within one year of diagnosis [56]. Unfortunately, there are currently no new antitumor agents demonstrating sufficient therapeutic success to significantly enhance the quality of life and life expectancy for these patients. Intriguingly, the Clinical Trials database (classic.clinicaltrials.gov) documents over a thousand clinical trials related to GBM treatment, with at least 20 trials investigating the potential of OVs from different families as therapeutic agents for both pediatric and adult patients. These viruses exhibit the capability to eliminate tumor cells with limited or no extratumoral toxicity, positioning them as highly effective oncolytic virotherapy (OVT) options that minimize systemic effects on the patient [57,58].

In the past decade, there has been a substantial increase in scientific publications proposing various viruses as antitumor agents [29,59]. Some OVs have even received FDA approval for treating different types of cancer. One of the main challenges for the use of OVs as OVT, besides viral safety, is that OVs are expected to exert a prolonged oncolytic effect, making an impact in cases of recurrence. This implies that the OV persists in healthy tissue for extended periods without causing disease in the host. This persistence facilitates the trafficking of viral particles to the tumor or metastatic lesions, increasing the likelihood of exerting oncolytic activity [60].

## 5. ZIKV Therapy in GBM

### 5.1. Mechanisms in CNS Tumors

Zika virus (ZIKV) is inherently a neurotropic pathogen, exhibiting a pronounced specificity for infecting neural progenitor cells (NPCs) [61] and healthy astrocytes [62]. Renowned for its high neurotropism, ZIKV demonstrates a broad and facile replication within cells of the CNS [63]. Intriguingly, both in vitro and in vivo models have shown a remarkable ability by ZIKV to infect glioma stem cells (GSCs), a distinct subset of cells within astroglial tumors of the CNS that share key characteristics with NPCs, including self-renewal and dedifferentiation [9] The establishment of molecular markers for virus recognition and cell entry, coupled with the disruption of various processes favoring cell death, has provided a foundational understanding for investigating ZIKV as an OV.

In the context of CNS tumors, ZIKV exhibits a remarkable degree of selectivity, targeting GSCs, which are known for their self-renewal capacity and resistance to conventional treatments like radiation and chemotherapy when in a dedifferentiated state, thus becoming a specific focus for ZIKV infection [64]. The heightened permissiveness of GBM to ZIKV has been strongly associated with the expression of the AXL receptor [65]. Moreover, the virus’s neurotropism appears to be correlated with the expression of SOX2 and the αVβ5 integrin, with particularly robust associations identified in GSCs [66]. Other study proposed that the expression level of MSI1 is implicated in ZIKV replication. The deficient expression of this protein in most healthy adult tissues, along with the expression and activation of the IFN-mediated antiviral response, restricts viral replication. In contrast, high MSI1 expression in tumors enhances replication. This underscores the high specificity of ZIKV infection toward tumors and the limited side effects in patients [67].

**Figure 2:**
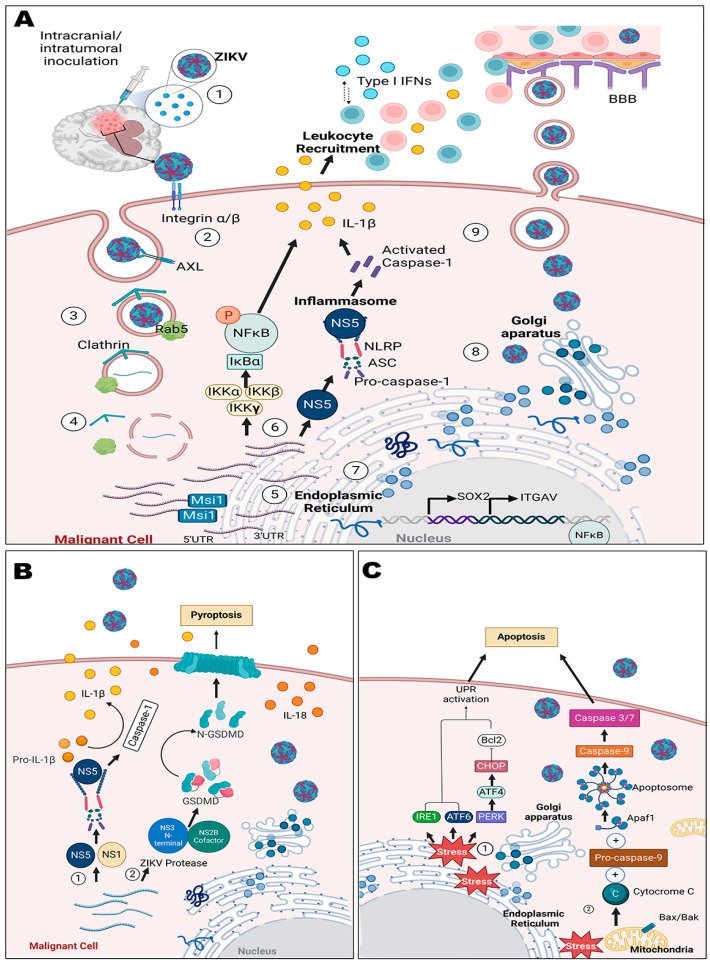
Oncolysis mechanism in GBM caused by ZIKV infection. ZIKV promotes a series of cytopathic and cytolytic events in tumor cells, as summarized in this image. Panel (**A**) (1). ZIKV is inoculated intracranially directly into the tumor or into the encephalic space, and then (2) viral particles are recognized by cell-surface receptors such as AXL and integrins and enter the malignant cell through clathrin-mediated endocytosis. Interestingly, SOX2, a transcription factor implicated in promoting tumorigenesis and cancer progression and found to be highly expressed in glioma stem cells, might indirectly mediate binding to ZIKV through the regulation of the ITGAV locus, which codes for integrin alpha V (or αv). (3). Early endosomes containing the viral particles are formed and trafficked via Rab5. (4). Viral RNA becomes uncoated and late endosomes disintegrate, liberating ZIKV genetic material into the cytoplasm and gets into the endoplasmic reticulum (ER). (5) Viral transcription and replication then occurs. Different proteins that are highly expressed in infected tumor cells can facilitate viral replication, e.g., Msi1 interacts with a binding element located in the 3′ UTR of the ZIKV genome, repressing translation initiation while keeping mRNA levels rising as more viral genomes replicate. (6). As it replicates, ZIKV induces different inflammatory responses via activation of the NLRP3 inflammasome through NS1 or NS5 proteins or via activation of the transcriptional regulators of the NF-kB/IkB family. Both pathways ultimately lead to the secretion of IL-1β, which facilitates the recruitment of T cells toward the microenvironment. T cells activate and secrete type I interferons (IFNs) to maintain host inflammatory responses. (7). Translation of viral proteins takes place in the ER, and immature viral particles are assembled. (8). Immature virions are transported into the Golgi apparatus to mature and then are released into the cytosolic space, either to persist in the infected cell and activate specific signals or (9) to be exported into the extracellular space, where they can either infect other neighboring cells in the tumor microenvironment or other brain cells; also, viral particles can cross the BBB into the systemic circulation. Panel (**B**). It has been proposed that during ZIKV infection, both caspase-1-dependent and -independent cleavage of GSDMD lead to pyroptosis. (1). Canonical caspase-1-dependent cleavage of GSDMD relies on the activation of the inflammasome and the release of caspase-1 to cleave GSDMD. (2). During caspase-1-independent cleavage of GSDMD, it is the ZIKV protease that effects the GSDMD cleavage. The cleaved N-terminal fragment of GSDMD oligomerizes into a ring-shaped structure that opens into membrane pores that lead to cell swelling and membrane rupture followed by cell lysis and the leaking of pro-inflammatory cytokines like interleukin-1β (IL-1β) and interleukin-18 (IL-18) into the extracellular space, as well as other highly inflammatory cellular contents. Panel (**C**). ZIKV infection of tumor cells can induce stress in membranes and mitochondria to trigger apoptosis. (1). In response to ER stress, many transmembrane proteins are activated in the ER membrane, such as IRE1 (inositol-requiring enzyme 1), ATF6 (activating transcription factor 6), and PERK (protein kinase R-like ER kinase). Each transmembrane protein works toward activating signaling cascades to unleash the unfolded protein response (UPR). The UPR is meant to restore ER homeostasis and alleviate ER stress, but if the stress is severe or prolonged and cannot be resolved, the UPR mechanisms lead to apoptosis. (2). Mitochondrial stress resulting from ZIKV infection leads to the release of cytochrome c into the cytoplasm of the cell. Cytochrome c induces the formation of the apoptosome, consisting of cytochrome c, Apaf-1 (apoptotic protease-activating factor 1), and procaspase-9. This complex activates procaspase-9, converting it into active caspase-9, which subsequently activates other downstream caspases, such as caspase-3 and caspase-7, to execute apoptosis. Image created using Biorender.com (last accessed on 29 January 2024).

Exploring the mechanisms underlying ZIKV’s oncolytic effects in in vitro and in vivo models, it has been suggested that the virus induces apoptosis and other forms of cell death, including caspase-independent pyroptosis. The latter has been reported to be induced by the viral protease activity of the NS2B3 non-structural viral protein, which specifically cleaves human gasdermin D (GSDMD) protein, releasing the N-terminal pore-forming domain to oligomerize and form pores on the cell membrane, thus leading to cell swelling, membrane rupture, and eventually cell lysis; however, there are some GSDMD variants that can be resistant to ZIKV cleavage or that are defective in oligomerizing the N-terminus GSDMD cleavage product, meaning that ZIKV tumor cytotoxicity depends on the patient’s GSDMD genetic background [68]. Nevertheless, this pyroptotic process contributes to the remodeling of the tumor microenvironment, enhancing the infiltrative response by CD8+ T cells [69] and promoting the upregulation of memory CD4+ T cells [70]. Figure 2 summarizes the above-mentioned processes.

Interestingly, a report from Iannolo et al. showed that ZIKV-infected GSC caused an increase in cell differentiation, induced caspase activity, and caused a significant increase in microRNA 34c (miR34c) expression. The Mir-34 family is involved on the regulation of different genes, such as Bcl2 (which participates in the inhibition of the apoptotic pathway) and NOTCH and NUMB (which are involved in stemness maintenance and in normal nervous system development). Overexpression of miR34c mimicked the effects observed in ZIKV infection. It induced differentiation in both GSCs and NSCs and had an impact on cell growth. Importantly, miR34c seemed to regulate both Bcl2 and NUMB expression, restricting the tumor cell’s ability to avoid apoptosis and potentially enhancing the effectiveness of chemo/radiotherapies for GBM treatment [71] and prompting miR34 family members to be considered as potential GBM therapy. However, more studies are needed to confirm this.

Although most available information focuses on the effect of ZIKV in GBM, a recent paper highlights ZIKV’s promising impact on neuroblastoma tumors, a prevalent extracranial malignant solid tumor often diagnosed in infants. Using an intratumoral method of virus introduction for optimal local delivery, Mazar et al. demonstrated permissive tumor infections in in vivo and ex vivo experimental subjects. This approach resulted in a rapid loss of tumor mass, with no recurrence even up to 4 weeks post-treatment, offering a remarkable survival advantage to the host (5- to 7-week-old female NCr nude athymic mice). This outcome correlates with the discovery of the association between CD24 expression and ZIKV sensitivity. CD24, abundant in all neuroblastoma tumors, appears to be a predictor of neuroblastoma cells’ permissiveness to ZIKV infection. These findings suggest the success of ZIKV as an OVT in neuroblastoma cells, opening a new avenue to address progenitor cells involved in various cancers, especially those expressing CD24 [72].

Lastly, the success of ZIKV as an OV seemed evident in a case report of a 43-year-old Brazilian woman with an infiltrative high-grade glioma confirmed as a GBM after tumor resection. After surgery, the patient had radiotherapy and chemotherapy with TMZ; however, a few weeks later she became infected with ZIKV. Six years after this, the patient remained in remission with no GBM recurrence despite carrying wild-type copies for the IDH1 and IDH2 genes together with mutations in oncogenes such as PIK3CA, clinically associated with patients who develop GBM at a young age and who had a poor survival prognosis [73]. The latter highlights the low toxicity that ZIKV seems to have in adults, which might support the clinical translation of WT ZIKV in controlled trials.

### 5.2. Cellular and Animal Models

Studies on ZIKV in animal models and in vivo settings have yielded promising results. Zhu et al. proposed a potential synergy between ZIKV and the TMZ chemotherapeutic agent in the treatment of GBM. Although the specific mechanism for this synergy is yet to be fully understood, the combination of ZIKV and TMZ presents an avenue for exploring ZIKV as a potential OVT alongside traditional approaches [8]. In an in vitro model, the oncolytic effects of WT ZIKV and a mutant-derived virus, ZIKV-E218A, were compared against three models of GSC. Both strains demonstrated tumoricidal effects, with the WT strain showing more potent effects. Further evaluation assessing the combined efficacy of TMZ and ZIKV-E218A showed that the conjugated use of these agents for one week exhibited greater antitumor efficacy and induction of apoptosis compared with their independent use [8].

On the other hand, ZIKV’s infection persistency is an important issue to understand. The results obtained in a Rhesus monkey model showed that ZIKV can persist in cerebrospinal fluid for up to 42 days after the primary infection [74]. Accordingly, Limonta et al. demonstrated that ZIKV infection in human fetal astrocytes can persist for several days due to a sustained antiviral response, suggesting a delicate balance between cell survival and viral persistence [75].

Different evidence postulates promising results for ZIKV as an OVT. For example, Chen et al. used female BALB/c nude mice with the orthotopic xenographic implantation of glioma stem cells or differentiated glioblastoma cells. Inoculation with an intracranial ZIKV vaccine resulted in a significant decrease in tumor size, improved survival, and no adverse effects on behavior, demonstrating the efficacy of a modified ZIKV in reducing brain masses and preventing animal death [76]. Kaid et al. performed intrathecal injection of 10^6^ PFU of the ZIKV-BR strain in three adult immunocompetent dogs with sporadic CNS cancer, which resulted in reduced tumor size, improved neurological symptoms, increased survival, and no observed adverse effects for at least 120 days [77]. Accordingly, Nair et al. developed an in vivo model using 8-week-old C57BL6/J mice with intraencephalic implantation of murine glioma cells (GL261 or CT2A). Fourteen days after tumor implantation, the mice were inoculated with 10^5^ plaque-forming units (PFU) of the ZIKV-Dakaral strain. The results demonstrated a reduction in tumor size 14 days after ZIKV treatment and protection against syngeneic tumor recurrence, surviving for at least 150 days, indicating a prolonged oncolytic effect [69]. Likewise, Ferreira et al. reported that mice with embryonal CNS tumors exhibited increased survival, a reduced tumor burden, and decreased metastasis capacity after infection with the Brazilian ZIKV-BR strain. Even after systemic administration of the virus, there were no neurological effects or adverse events observed in other organs [78].

Despite all the above-mentioned information, currently there are no clinical trials for ZIKV as an OVT; to our knowledge, all studies are still in a pre-clinical phase. This is probably due to several critical aspects that remain unclear, such as control mechanisms, the persistence of infection, potential secondary inflammation of the CNS, and mechanisms preventing relapse. These factors are crucial for the safe and enduring application of ZIKV as an OVT. Nevertheless, a Phase 1 clinical trial using two ZIKV strains in controlled human infection models is currently ongoing (NCT05123222), with the purpose of evaluating the clinical and virologic response to escalating doses of ZIKV in healthy male and non-pregnant, female adult volunteers. These results will then be used to evaluate the protective efficacy of candidate ZIKV vaccines prior to evaluation of these candidates in Phase 2 clinical trials [79]. It is possible that these results open an avenue to study the doses of wild-type ZIKV strains needed to cause effects on different tumoral cells.

Further studies are necessary to determine whether ZIKV treatment enhances the functional responses of antitumor T cells against GSCs or engages other immunomodulatory mechanisms. Additionally, investigations into cellular and molecular mechanisms beyond those discussed herein may also play a role in the oncolytic activity of the virus. Exploring the impact of ZIKV OVT in conjunction with other therapeutic approaches is also essential for a comprehensive understanding and for potential clinical applications.

## 6. Conclusions

The results derived from extensive in vitro and in vivo studies strongly support the oncolytic potential of various strains of ZIKV, particularly in the context of CNS tumors, with a pronounced focus on high-grade gliomas. Furthermore, the application of ZIKV has demonstrated safety in both murine and canine animal models, providing a solid foundation for clinical exploration through controlled trials to evaluate its efficacy as an OVT in human subjects. However, the journey toward clinical application requires further investigations covering diverse treatment protocols, optimal virus administration schemes, dosage refinement, comprehensive long-term safety assessments, and potential synergies with other pharmaceuticals or therapeutic alternatives. With its neurotropic and oncolytic capabilities, ZIKV stands out as a valuable viral agent, presenting a promising and effective alternative for the treatment of individuals globally diagnosed with high-grade astroglial brain tumors. The continuous exploration of ZIKV’s therapeutic potential holds immense promise for the advancement of glioblastoma multiforme treatment strategies.

## Figures and Tables

**Table 1:** Completed or active (recruiting and non-recruiting) clinical trials for OVs in GBM.

OV		Type	Description	Phase	Type of CNS Tumor	CLT ID and/or References
AdV	Ad-TD-nsIL12	Eng	Deletion of E1ACR2, E1B19K and E3gp19K, WT E3B; armed with nsIL12, signal peptide deleted	I	Primary and progressive pediatric diffuse intrinsic pontine glioma	NCT05717712; NCT05717699 [40,41,42]
	DNX-2401 (also known as tasadenoturev; Delta-24-RGD)	Eng	24 bp deletion in E1A; RGD peptide insertion into the fiber knob that allows the virus to anchor directly to integrins	I, II	Naive diffuse intrinsic pontine gliomas	NCT03178032; NCT01956734; NCT02197169 [43]
	ADV-TK	Eng	Vector contains the TK gene	I	Recurrent high-grade glioma	NCT00870181 [44]
	DNX-2440	Eng	24 bp deletion in E1A; insertion of OX40L and RGD-4C genes	I	First or second recurrence of GBM	NCT03714334
HSV-1	G207	Eng	Deletion of γ134.5 gene, insertion of lacZ operon in UL39	I, II	Recurrent or refractory cerebellar brain tumors; progressive or recurrent supratentorial brain tumors; recurrent high-grade glioma; malignant glioma	NCT03911388; NCT02457845; NCT04482933; NCT00157703; NCT00028158 [45]
	MVR-C5252	Eng	Active domain of human IL-12 and Fab fragment of anti-PD-1 antibody	I	Recurrent high-grade glioma	NCT06126744 [46]
	rQNestin (also known as rQNestin34.5v.2)	Eng	Restoration of one copy of ICP34.5 under transcriptional control of NP/EE	I	Malignant glioma	NCT03152318 [47]
	M032	Eng	a γ134.5-deleted HSV-1 engineered to express murine IL-12	I	Recurrent malignant glioma	NCT02062827 [48]
Poliovirus	PVS-RIPO (also known as Lerapolturev)	Eng	Recombinant, non-pathogenic poliovirus:rhinovirus chimera; genome of PV1S with its cognate IRES element replaced with that of HRV2	I, II	Malignant glioma (primary and recurrent); GBM	NCT02986178; NCT03043391; NCT01491893; NCT04479241
REO	Reolysin	WT	Strain of a type 3 REO that selectively infects and lyses tumor cells via the Ras-activated pathway	I, II	Recurrent malignant gliomas; with high-grade relapsed or refractory brain tumors	NCT00528684; NCT02444546
Retrovirus	Toca511 (also known as Vocimagene amiretrorepvec)	Eng	Non-lytic RRV that delivers a yeast cytosine deaminase to convert the prodrug Toca FC into the antimetabolite 5-fluorouracil	I, II, III	Recurrent brain tumors (anaplastic astrocytoma; anaplastic oligoastrocytoma; anaplastic oligodendroglioma; GBM)	NCT01985256; NCT01470794; NCT01156584
MV	MV-CEA	Eng	CEA incorporated in the vector	I	Recurrent GBM	NCT00390299
Vaccinia virus	TG6002	Eng	Vaccinia virus strain Copenhagen with deletion of TK and RR genes, expresses the FCU1 gene	I	Recurrent GBM	NCT03294486 [49]
Parvovirus	ParvOryx	WT	WT rat parvovirus H1 (H-1PV)	I, II	Progressive primary or recurrent GBM	NCT01301430 [50]

Table abbreviations are disclosed at the bottom of the article.

## Data Availability

No new data were created or analyzed in this study. Data sharing is not applicable to this article.

## Abbreviations

AdV	adenovirus
bp	base pair
BBB	blood–brain barrier
CEA	carcinoembryonic antigen
CNS	central nervous system
CSC	cancer stem cells
CLT	Clinicaltrials.gov ID
EGFR	epidermal growth factor receptor
EMA	European Medicines Agency.
Eng	engineered
ER	endoplasmic reticulum
FDA	United States Food and Drug Administration Agency
GBM	glioblastoma multiforme
GBM-I	glioblastoma multiforme type 1
GBM-II	glioblastoma multiforme type 2
GBR	glioblastoma recurrence
GFAP	glial fibrillary acidic protein
GSDMD	gasdermin D protein
GSC	glioma stem cells
HR2V	human rhinovirus type 2
HSV	herpes simplex virus
IDH1	isocitrate dehydrogenase type 1 enzyme
IDH2	isocitrate dehydrogenase type 2 enzyme
IFN	interferon
IL-1β	interleukin-1β
IL-18	interleukin-18
IRES	internal ribosomal entry site
MGMT	O-6-Methylguanine-DNA-methyltransferase gene
MV	measles virus
mRNA	messenger RNA
ncRNA	non-coding RNAs
NPC	neural progenitor cells
NP/EE	Nestin Promoter/Enhancer Element
NK	natural killer cells
nsIL-12	non-secretory interleukin-12
OVT	oncolytic virotherapy
OV	oncolytic virus
PDGF	platelet-derived growth factor
PDGFRα	receptor alpha of the PDGF
PTEN	phosphatase and tensin gene
PVS1	live attenuated poliovirus serotype 1 (SABIN) vaccine
RGD	specific amino acid sequence Arg-Gly-Asp
REO	reovirus
RR	ribonucleotide reductase
RRV	retroviral replicating vector
RV	rotavirus
TK	thymidine kinase
TSGs	tumor suppressor genes
T-VEC	Talimogene laherparepvec
TMZ	Temozolomide
VEGF	vascular endothelial growth factor
WHO	World Health Organization
WT	wild-type
ZIKV	Zika virus
